# SAG/RBX2 E3 ligase complexes with UBCH10 and UBE2S E2s to ubiquitylate β-TrCP1 via K11-linkage for degradation

**DOI:** 10.1038/srep37441

**Published:** 2016-12-02

**Authors:** Peng Kuang, Mingjia Tan, Weihua Zhou, Qiang Zhang, Yi Sun

**Affiliations:** 1 Department of Internal Medicine, Beijing University School of Medicine, 38 Xueyuan Road, Beijing, 100191, China; 2 Division of Radiation and Cancer Biology, Department of Radiation Oncology, University of Michigan, 4424B MS-1, 1301 Catherine Street, Ann Arbor, MI 48109, USA; 3 Institute of Translational Medicine, Zhejiang University School of Medicine, Hangzhou, Zhejiang, People’s Republic of China; 4 Collaborative Innovation Center for Diagnosis and Treatment of Infectious Diseases, Zhejiang University, Hangzhou, P. R. China

## Abstract

SAG/RBX2 and RBX1 are two family members of RING components of Cullin-RING ligases (CRLs), required for their enzymatic activity. Previous studies showed that SAG prefers to bind with CUL5, as well as CUL1, whereas RBX1 binds exclusively to CULs1–4. Detailed biochemical difference between SAG and RBX1, and whether SAG mediates cross-talk between CRL5 and CRL1 are previously unknown. Here we report that the levels of SAG and β-TrCP1 are inversely correlated, and SAG-CUL5-βTrCP1 forms a complex under physiological condition. SAG-CUL5, but not RBX1-CUL1, negatively modulates β-TrCP1 levels by shortening its protein half-life through promoting its ubiquitylation via atypical K11-linkage. Consistently, chemical inducers of SAG reduced β-TrCP1 level. Furthermore, SAG mainly binds to E2s UBCH10 and UBE2S known to mediate K11 linkage of ubiquitin, whereas RBX1 exclusively binds to E2s CDC34 and UBCH5C, known to mediate K48 linkage of ubiquitin. Finally, silencing of either UBCH10 or UBE2S, but not UBCH5C, caused accumulation of endogenous β-TrCP1, suggesting that β-TrCP1 is a physiological substrate of SAG-UBCH10C/UBE2S. Our study, for the first time, differentiates SAG and RBX1 biochemically via their respective binding to different E2s; and shows a negative cross-talk between CRL5 and CRL1 through SAG mediated ubiquitylation of β-TrCP1.

Protein ubiquitylation is a post-translational modification, that via modulating protein stability, activity, or localization^1^ regulates many cellular pathways including proinflammatory signaling, DNA damage response, and apoptosis^2^,^3^. Protein ubiquitylation is catalyzed by an E1 ubiquitin-activating enzyme, an E2 ubiquitin-conjugating enzyme, and an E3 ubiquitin ligase, that is responsible for substrate recognition^4^, and catalyzes the transfer of ubiquitin directly from the E2 to the substrate^5^. Multiple run of this cascade reaction results in polyubiquitylation of a substrate^6^. Such ubiquitin chains can be connected through the N-terminus of ubiquitin or through one of its seven lysine residues, leading to assembly of diverse polyubiquitin chains with different topologies for distinct structures and functions^7^. While K63-linked chains are mostly implicated in proinflammatory signaling, K48-linked polyubiquitin chains predominantly target proteins for proteasomal degradation^8^. K11-linked chains have been less studied than K48 or K63 linkages, but they seem to serve as a degradation signal for APC/C substrates in the regulation of cell division^9^,^10^.

The mechanisms of linkage specificity in polyUb chain synthesis by E2s and E3s are not well understood and remain an area of active investigation. Unlike HECT E3 ligases, which possess an active-site cysteine that receives Ub from a charged E2 (E2~Ub) and subsequently transfers it onto a substrate lysine, the RING ligases lack a catalytic cysteine and act instead by bringing the substrate lysine and catalytic cysteine of E2~Ub together in a conformation suitable for Ub transfer. Thus, E2s of RING ligases determine which polyUb linkage(s) will be synthesized. It has been established that CDC34 or UBCH5C E2s couples with CRL1, also known as SCF (SKP1-Cullin1-F box protein) E3, to assemble the ubiquitin chain via the K48 linkage^11^, whereas UBCH10/UBE2C and UBE2S couples with APC/C (Anaphase Promoting Complex/cyclosome) E3 to assemble the ubiquitin chain via the K11 linkage^12^, both are for targeted degradation of their respective substrates. It is, however, totally unknown whether and how CRLs would couple with UBCH10/UBE2C and UBE2S to assemble the ubiquitin chain via the K11 linkage.

In humans, there are only two family members of RING components of CRLs, RBX1 and RBX2^13^. RBX1 (also known as ROC1) is constitutively expressed and preferentially bound to CUL1–4, whereas SAG/RBX2 (also known as ROC2) is stress inducible with preferential association with CUL5^14^ as well as CUL1^15^. Both proteins are evolutionarily conserved^13^,^16^, but functionally non-redundant during mouse embryonic development, given that germline knockout of either *Rbx1* or *Sag* causes embryonic lethality^15^,^17^. On the other hand, it appears that their E3 ligase activity is biochemically interchangeable in carrying out an *in vitro* polyubiquitylation reactions when RBX1 or SAG, used as the source of E3, was purified from transfected cells through immunoprecipitation^18^,^19^. It is, however, totally unknown whether SAG and RBX1 bind to different E2s to assemble different linkage of ubiquitin chains. Still unknown is whether and how a cross-talk exists between CRL1 and CRL5 mediated by SAG.

In this study, we report that β-TrCP1, a well-studied F-box protein^20^, binds to both CUL1 and CUL5, and is subjected to ubiquitylation and degradation by SAG-CUL5 or SAG-CUL1, but not RBX1-CUL1 E3 ligase. We also found that SAG-CUL5-mediated ubiquitylation of β-TrCP1 is via the K11 linkage, achieved by SAG binding to K11 linkage E2s, UBCH10/UBE2C and UBE2S, and silencing of either UBCH10 or UBE2S caused β-TrCP1 accumulation. Our study, therefore, revealed, for the first time, that there is a negative cross-talk between CRL1 and CRL5 through SAG-mediated ubiquitylation and degradation of β-TrCP1, and that CRL5 E3 can mediate K11-linked ubiquitin chain through SAG binding to specific E2s.

## Results

### A cross-talk between SAG and β-TrCP1

**Figure 1:**
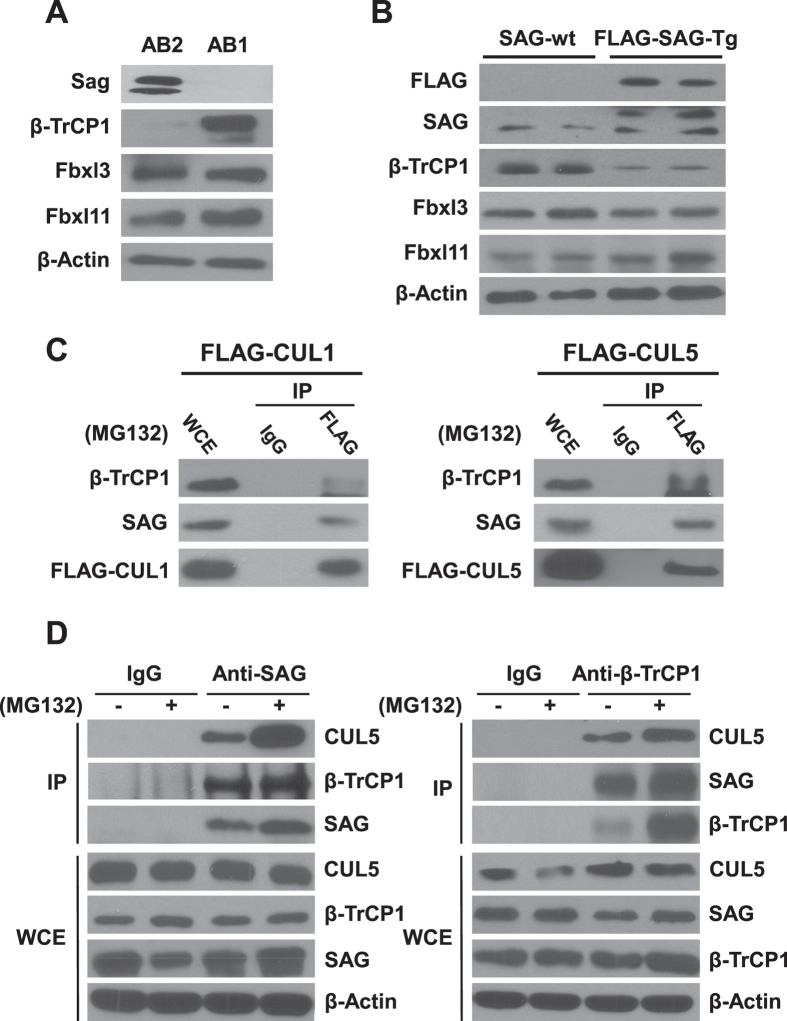
Inverse relationship between SAG and β-TrCP1, and their binding to cullins. (**A** and **B**) The levels of SAG and β-TrCP1 are inversely correlated: Cell lysates were prepared from mouse embryonic stem cells (AB2, wild type and AB1, Sag-null)^19^ (**A**) or mouse pancreatic tissues (**B**), and subjected to immunoblotting with indicated Abs. (**C** and **D**) SAG-β-TrCP1-cullins form the complex: The 293 T cells were transfected with FLAG-tagged CUL1 or CUL5, followed by IP with anti-FLAG Ab or IgG control, and IB with indicated Abs (**C**). A549 cells were left untreated or treated with 10 μM MG132 for 4 hrs prior to harvesting. Whole cell extracts (WCE) were immunoprecipitated with antibodies against SAG or β-TrCP, along with IgG control, followed by immunoblotting with indicated antibodies (**D**).

During our study of the effect of *Sag* deletion on the differentiation of mouse embryonic stem cells (mESC)^15^, we unexpectedly found an inverse relationship between SAG and β-TrCP1. In Sag-null mES AB1 cells, we observed a remarkable accumulation of β-TrCP1, as compared to the wild type mES AB2 cells (Fig. 1A). Consistently, we found a significant reduction of β-TrCP1 levels in pancreatic tissues expressing transgenic SAG protein (manuscript in preparation) (Fig. 1B). The Sag-mediated β-TrCP1 changes appeared to be rather specific, since no changes were found in FBXL3 and FBXL11, two F-box proteins upon SAG manipulation (Fig. 1A and B). Following this lead, we determined the levels of SAG and RBX1 in comparison with those of three F-box proteins in multiple lung cancer cell lines and found in general that the levels of SAG, but not of its family member RBX1, is correlated in an inverse manner with the levels of β-TrCP1 only (Fig. S1A). Thus, SAG appears to negatively regulate β-TrCP1.

Since SAG is known to preferentially complex with CUL5^14^ through its N-terminal domain to the C-terminal domain of CUL5^21^, whereas β-TrCP1 is known to bind to CUL1 to form CRL1^22^, we first determined potential binding between β-TrCP1 and two cullins. Indeed, in an immunoprecipitation assay, endogenous β-TrCP1 as well as SAG were pulled down by either CUL1 or CUL5 (Fig. 1C), whereas RBX1 was pulled down only by CUL1, but not by CUL5 (Fig. S1B). We further determined endogenous binding of among SAG, CUL5, and β-TrCP1 and found this tri-complex does exist using a pull down assay with either anti-SAG or anti-β-TrCP1 antibody, respectively (Fig. 1D). Moreover, β-TrCP1-SAG binding was significantly enhanced when protein degradation was inhibited by MG132 (Fig. 1D). Thus, β-TrCP1 forms a complex with SAG-CUL5 under non-stressed physiological conditions.

### SAG-CUL5, but not RBX1-CUL1 reduces the β-TrCP1 level via shortening its protein half-life

**Figure 2:**
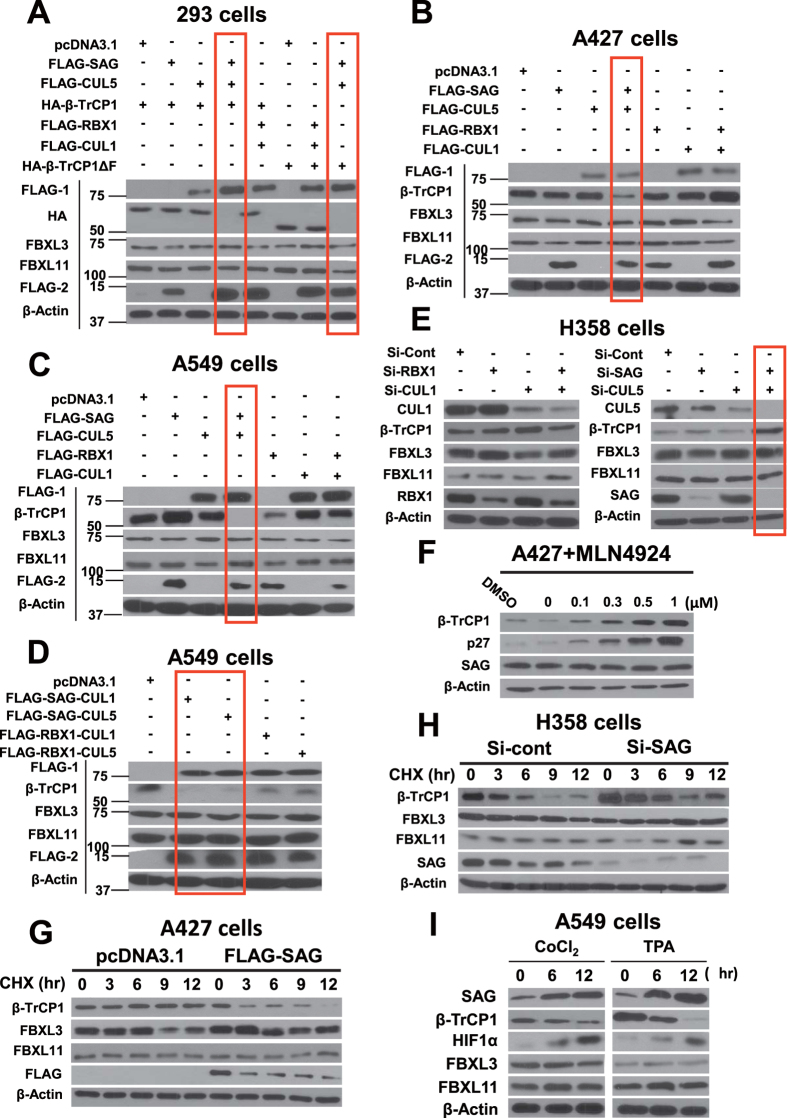
SAG negatively regulates β-TrCP1 protein level. (**A**–**D**) SAG negatively regulates β-TrCP1 protein level: Various cell lines, as indicated were transfected with indicated plasmids alone or in combination. Whole cell extracts were analyzed by immunoblotting with indicated antibodies. FLAG-1: CUL1 or CUL5; FLAG-2, SAG or RBX1. β-TrCP1 accumulation upon silencing of SAG and CUL5: H358 cells were transfected with siRNAs targeting SAG, RBX1, CUL1 or CUL5. Whole cell extracts were analyzed by immunoblotting with indicated antibodies. Accumulation of β-TrCP1 and p27: A427 cells were treated with indicated concentrations of MLN4924 for 24 hrs, followed by immunoblotting using indicated Abs. (**G** and **H**) SAG manipulates protein half-life of β-TrCP1: A427 or H358 cells were either transfected with FLAG-SAG (**G**) or si-SAG (**H**) for 12 hrs. Cells were then cultured in fresh medium containing CHX and incubated for indicated time periods before being harvested for immuoblotting with indicated Abs. SAG induction reduces β-TrCP1 level: A549 cells were treated with CoCl_2_ (250 μM) or TPA (20 ng/mL) for indicate periods of time, followed by immunoblotting analysis using indicated Abs.

Given that SAG-CUL5 form active CRL5 E3 ubiquitin ligase, we tested our hypothesis that β-TrCP1 could be a novel substrate of CRL5. We, therefore, determined whether β-TrCP1 protein level is negatively regulated by SAG or CUL5, alone or in combination, as compared to RBX1 and CUL1 in combination. In a co-transfection experiment in 293 cells, transfection of neither SAG nor CUL5 alone had any significant effect on the levels of ectopically expressed β-TrCP1. Interestingly, when transfected in combination, SAG-CUL5, but not RBX1-CUL1, significantly reduced the levels of ectopically expressed β-TrCP1 as well as β-TrCP1ΔF, a ligase-dead dominant negative mutant^23^ (Fig. 2A), excluding the possibility of degradation via self-ubiquitylation. Combination of SAG-CUL5 had no effect on the levels of FBXL3 and FBXL11 (Fig. 2A). We further determined which protein, among SAG, RBX1 CUL5 and CUL1, is responsible for mediating β-TrCP1 reduction and found that SAG, in combination with either CUL5 or CUL1, is able to reduce β-TrCP1 level, whereas RBX1 had no effect regardless its combination with CUL1 or CUL5 (Fig. S2A). Thus, SAG plays a critical role in negative regulation of β-TrCP1.

We next determined the effect of transfected SAG/CUL5 or RBX1/CUL1 on endogenous levels of β-TrCP1 in two lung cancer cell lines, A427 and A549 harboring relatively high levels of β-TrCP1 (Fig. S1A). Transfection of either SAG or CUL5 had moderate, if any, effect, but the combination of SAG-CUL5, not of RBX1-CUL1 remarkably reduced the levels of β-TrCP1 without affecting FBXL3 and FBXL11 (Fig. 2B and C). Again, SAG in combination with either CUL1 or CUL5 caused completely elimination β-TrCP1, whereas RBX1 had a minimal effect, if any, regardless its combination with CUL1 or CUL5 (Fig. 2D).

We then used loss-of-function approaches via either genetic siRNA knockdown or pharmacological small molecule inhibitor to further determine the role of SAG or RBX1 in combinations of CUL1 or CUL5 for β-TrCP1 targeting. Knockdown of SAG, CUL5, RBX1 or CUL1 alone or in combination was performed in H358 cells, which expressed a relatively low level of β-TrCP1 (Fig. S1A). A significant accumulation of endogenous β-TrCP1, but not FBXL3 or FBXL11, was observed only when both SAG and CUL5 were silenced simultaneously (Fig. 2E), indicating that β-TrCP1 is selectively degraded by SAG-CUL5, but not by RBX1-CUL1. We then used MLN4924, a small molecule inhibitor of NAE (NEDD8-Activating Enzyme), which indirectly inhibits the entire CRL E3 ligases by blocking cullin neddylation^24^, and found a dose-dependent accumulation of β-TrCP1 in all three lung cancer cell lines tested (Figs 2F and S2B,C), further supporting the notion that β-TrCP1 is a novel substrate of CRLs. A dose-dependent accumulation of p27, serving as a positive control, was also observed (Fig. 2F and S2B,C).

Given SAG-CUL5 is a typical E3 ubiquitin ligase, we, therefore, determined whether SAG-CUL5, but not RBX1-CUL1 would shorten the protein half-life of β-TrCP1. Indeed, in a co-transfection experiment, SAG-CUL5, but not RBX1-CUL1, shortened the protein half-life of transfected HA-β-TrCP1 (Fig. S2D). SAG-CUL5 also shortened the protein half-life of HA-β-TrCP1ΔF (Fig. S2E). Likewise, the protein half-life of endogenous β-TrCP1 was also significantly shortened upon SAG transfection into A427 cells which expressed high β-TrCP1 but low SAG (Fig. 2G). Consistently, siRNA knockdown of SAG caused the accumulation of basal level β-TrCP1 and extended the protein half-life of endogenous β-TrCP1 in H358, which expressed high SAG, but low β-TrCP1 (Fig. 2H). More importantly, we determined whether CoCl_2_ and TPA, two chemical agents known to induce SAG in our previous studies^25^,^26^ would reduce β-TrCP1 level. Indeed, a time dependent increase of SAG was accompanied by a time-dependent decrease of β-TrCP1, whereas increased SAG levels had no effect on FBXL3 and FBXL11, two F-box proteins not being regulated by SAG, serving here as negative controls (Fig. 2I). Finally, we excluded possibility that β-TrCP1 reduction by SAG-CUL5 occurred at the transcriptional level, since β-TrCP1 mRNA levels did not change regardless of overexpression or knockdown of SAG-CUL5 or RBX1-CUL1 alone or in combination (Fig. S2F). Taken together, our results support the notion that β-TrCP1 is a novel substrate of SAG-CUL5 E3 ligase.

### SAG-CUL5, but not RBX1-CUL1 promotes the ubiquitylation of β-TrCP1 via K11 linkage

To further confirm that β-TrCP1 is indeed a new substrate of SAG-CUL5 E3 ligase, we determined whether βTrCP1 is subjected to ubiquitylation by SAG-CUL5.

**Figure 3:**
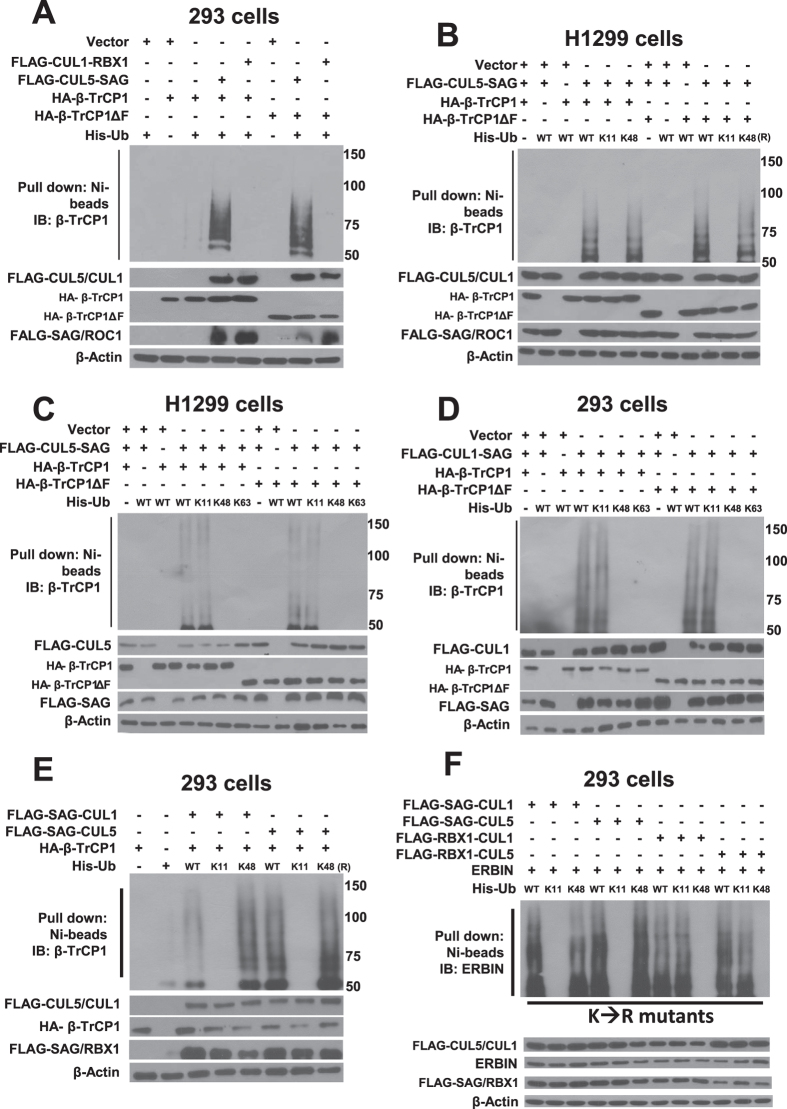
SAG-CUL5, but not RBX1-CUL1, promotes the ubiquitylation of β-TrCP1 via K11 linkage. SAG-CUL5 promotes polyubiquitylation of β-TrCP1 and β-TrCP1ΔF *in vivo*: 293 T cells were transfected with indicated plasmids, lysed under denatured condition by 6 M guanidinium solution, followed by Ni-bead pull-down. Washed beads were boiled and subjected to immunoblotting, along with whole cell lysates, using indicated Abs. **(B–F)** SAG/CUL5 promotes poly-ubiquitylation of β-TrCP1 via K11 linkage: H1299 (**B**) or 293 T (**C–F**) cells were cotransfected with indicated plasmids alone or in combination, along with ubiquitin and various ubiquitin mutants. Whole cell extracts and Ni-NTA affinity purified fractions were analyzed by immunoblotting using indicated Abs.

A cell-based *in vivo* ubiquitylation assay was performed where ubiquitylated proteins were captured with nickel nitrilotriacetic acid (Ni-NTA) affinity chromatography from 293 cells transfected with FLAG-RBX1-CUL1 or FLAG-SAG-CUL5, or the vector control, along with His-ubiquitin and HA-βTrCP1, followed by detection of ubiquitylated β-TrCP1 using anti-β-TrCP1 antibody (for both exogenous and endogenous proteins). Indeed, SAG-CUL5, but not RBX1-CUL1, promoted substantial polyubiquitylation of β-TrCP1 as well as β-TrCP1ΔF (Fig. 3A). The same result was observed when H1299 cells were used for transfection (Figure S3A). Thus, SAG-CUL5 promotes β-TrCP1 ubiquitylation.

We then examined the type of ubiquitin chain linkage for ubiquitylated β-TrCP1, using various ubiquitin mutants with a single K → R substitution. We found that polyubiquitylation chain of β-TrCP1 or β-TrCP1ΔF, catalyzed by SAG-CUL5, can be formed when ubiquitin used was wild type, K48R or K63R mutant, but not K11R mutant (Figs 3B and S3B), suggesting that it is likely through a K11 linkage. We further confirmed this finding by using another set of ubiquitin mutants in which all seven lysine (K) residues were mutated to arginine (R), except one indicated K residue remaining as wild type. Consistent with above observation, polyubiquitylation chain of β-TrCP1 or β-TrCP1ΔF can be formed when ubiquitin used was wild type or K11 mutant, but not other K48 or K63 mutant (Figs 3C and S3C). Moreover, we co-transfected SAG-CUL1, followed by *in vivo* ubiquitylation assay, to determine whether the K11 linkage is SAG specific. Indeed, SAG-CUL1 can also promote polyubiquitylation of β-TrCP1 and β-TrCP1ΔF when wild type or K11 ubiquitin mutant, but not other mutants, was used (Fig. 3D and S3D). Likewise, SAG, in combination with either CUL1 or CUL5, promotes polyubiquitylation of β-TrCP1 or β-TrCP1ΔF when wild type or K48R, but not K11R ubiquitin was used (Fig. 3E). These results clearly demonstrated that SAG, but not CUL1 or CUL5, plays the key role in promoting the K11 linkage.

Our previous^27^ and recent^28^ studies showed that p27 and Erbin are substrates of SAG E3 ligase. Here we determined the type of ubiquitin chain linkages catalyzed by different combination of SAG/RBX1 with CUL1/CUL5, and found that in both ERBIN and p27 cases, SAG promotes the ubiquitylation through the K11 linkage, whereas RBX1 promotes ubiquitylation via the K48 linkage, in a manner independent of cullins (Fig. 3F and S3E). Taken together, these *in vivo* ubiquitylation assays clearly demonstrated that SAG and RBX1, two RING components of CRL E3, determine the ubiquitin chain linkage via K11 or K48, respectively.

### SAG and RBX1 complex with different E2 enzymes to facilitate the formation of K11 or K48-linked ubiquitylation chain

**Figure 4:**
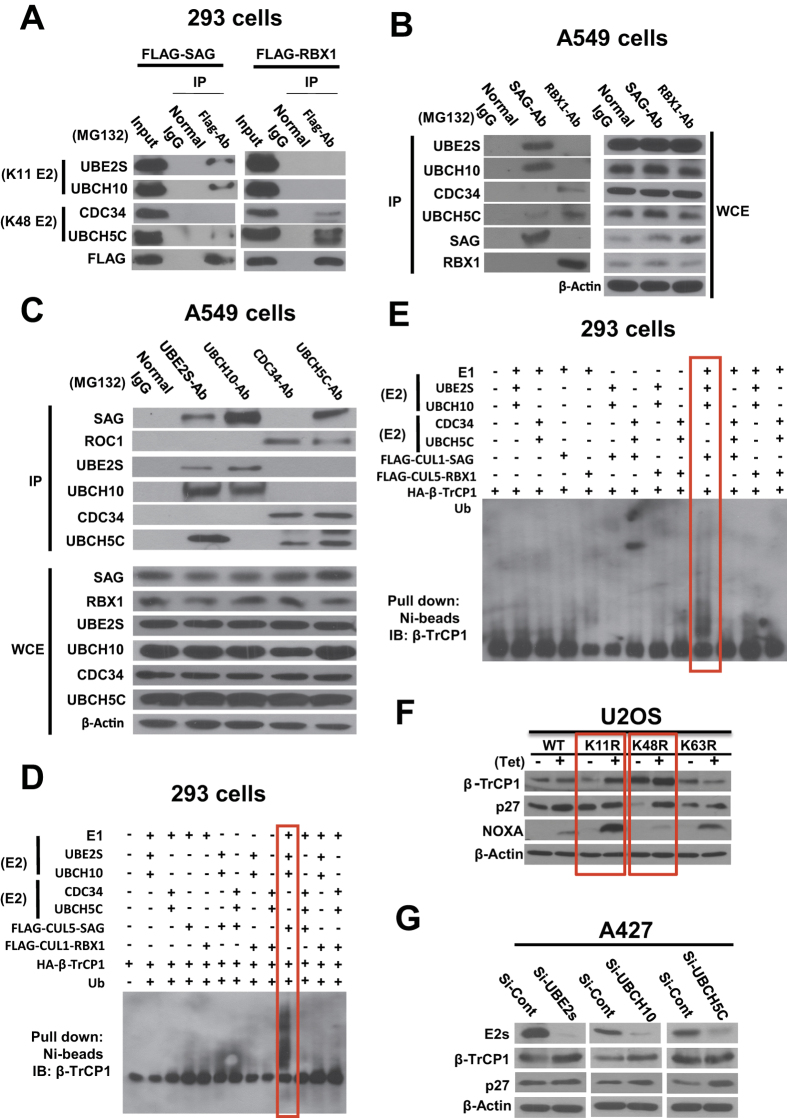
SAG and RBX1 form the complex with different E2s and β-TrCP is a substrate of SAG-UBCH10/UBE2S for targeted ubiquiylation via K11 linkage. The 293 T cells were transfected with FLAG-SAG or FLAG-RBX1, followed by IP using FLAG Ab or normal IgG control, and immunoblotting with indicated Abs. **(B** and **C)** Whole cell extracts (WCE) from A549 cells were subjected to immunoprecipitation and immunoblotting, or directly subjected to immunoblotting with indicated Abs. (**D** and **E**) The 293 T cells were transfected with indicated plasmids alone or in combination, and subjected to *in vitro* ubiquitylation as described in M&M. The reaction mixture was then loaded onto PAGE gel for immunoblotting using anti-β-TrCP Ab. **(F)** U2OS-shUb-Ub (WT), -Ub (K11R), -Ub (K48R), and -Ub (K63R) cells were treated with or without tetracycline (1 mg/mL) for 4 days before cell lysates were prepared for immunoblotting with indicated Abs. **(G)** A427 cells were transfected with siRNAs targeting UBCH10, UBE2S, or UBCH5C, along with scrambled controls. Cells were harvested 48 hr later and subjected to immunoblotting using indicated Abs.

It has been recently reported that UBCH10 (also known as UBE2C) and UBE2S are E2 conjugating enzymes, specifically for K11 linkage catalyzed by APC/C E3 ligase^12^,^29^. We, therefore, determined potential binding of SAG with UBCH10 or UBE2C, as well as RBX1 with UBCH5C or CDC34, two known E2s for K48 linkage^30^,^31^. Indeed, we found that ectopically expressed FLAG-SAG readily pulled down endogenous UBE2S and UBCH10 as well as trace amount of UBCH5C, but not at all CDC34, whereas ectopically expressed FLAG-RBX1 pulled down endogenous K48 E2 UBCH5C and CDC34, exclusively (Figs 4A and S4A). The endogenous binding of these E2–E3 pairs was further confirmed in two lung cancer cell lines under physiological unstressed conditions in a pull-down assay using antibody against either SAG or RBX1 (Figs 4B and S4B) or antibodies against each of four E2s (Figs 4C and S4C). Interestingly, two K11 E2s can bind to each other, whereas two K48 E2s bind to each other, but there is essentially no cross-binding among these E2s, except a weak binding between UBE2S and UBCH5C, likely mediated via SAG (Figs 4C and S4C). Binding of SAG to both K11 and K48 E2s may suggest that SAG could mediate polyubiquitylation via K48 linkage in a subset of substrates, in addition to K11 linkage. Alternatively, SAG may mediate a mixed K48 and K11 linkage.

Finally, we used *in vitro* ubiquitylation assay to determine polyubiquitylation of β-TrCP1, catalyzed by different combinations of E2/E3. We found that polyubiquitylation of βTrCP1 was found only when UBE2S and UBCH10 were in combination with either SAG-CUL5 (Fig. 4D) or SAG-CUL1 (Fig. 4E). Neither RBX1-CUL1 nor RBX1-CUL5 was able to promote β-TrCP1 polyubiquitylation, regardless which pair of E2s was used (Fig. 4D and E). In the case when ERBIN was used as the substrate, both K11 E2s (UBE2S/UBCH10) in combination with SAG-CUL5 E3, and K48 E2s (CDC34/UBCH5C) in combination of RBX1-CUL1 E3, promoted its polyubiquitylation (Fig. S4D). Taken together, our data indicate that through selective binding to K11 E2s UBE2S and UBCH10, SAG promotes poly-ubiquitylation of βTrCP1 as well as other known substrates, Erbin and p27 via K11 linkage, whereas RBX1 binds exclusively to K48 E2s CDC34 and UBEH5C to promote the polyubiquitylation of p27 and Erbin via K48 linkage.

### β-TrCP1 is mainly accumulated in U2OS-shUb-Ub (K11R) cells

To further demonstrate whether β-TrCP1 is degraded solely or mainly via K11 linkage at the cellular level, we detected β-TrCP1 expression in U2OS cells where endogenous ubiquitin was deleted and replaced with K11R, K48R and K63R mutants, respectively, using a tetracycline-inducible system^32^. We have validated this cellular system recently and found that it worked efficiently with wild type ubiquitin knockdown and mutant ubiquitin replacement upon tetracycline treatment^34^. It is anticipated that upon exposure to tetracycline, a given substrate will be accumulated in cells expressing a particular ubiquitin mutant, if that substrate is ubiquitylated via that particular linkage for degradation. Using this system, we found that β-TrCP1 was accumulated at the highest level in tetracycline-inducible K11R cells (Fig. 4F, lanes 3 vs. 4). Interestingly, a moderate β-TrCP1 accumulation was also found in tetracycline-inducible K48R cells (Fig. 4F. lanes 5 vs. 6), suggesting that it is likely that β-TrCP1 is subjected to ubiquitylation and degradation via both K11 and K48 linkage, although mainly via K11 linkage. Serving as the controls, NOXA is mainly ubiquitylated via K11 linkage for degradation, as we reported recently^33^, whereas p27 is solely ubiquitylated via K48 linkage for targeted degradation under this experimental conditions (Fig. 4F).

### β-TrCP1 is a physiological substrate of SAG-UBCH10/UBE2S

We next determined whether two SAG-binding, K11-linked E2s indeed play a role in SAG-mediated ubiquitylation and degradation of β-TrCP1. We, therefore, individually silenced UBCH10 or UBE2S, as well as K48-linked E2 UBCH5C as a control, to determine whether endogenous β-TrCP1 would be accumulated and under which knockdown conditions. As shown in Fig. 4G, silencing either UBCH10 or UBE2S, but not UBCH5C caused β-TrCP1 accumulation, whereas silencing UBCH5C, but not UBCH10 nor UBE2S, caused accumulation of p27, a protein whose ubiquitylation is mediated mainly by K48 linkage, although under overexpressed condition, SAG-CUL5 did promote its ubiquitylation via K11 linkage in 293 cells (Fig. S3E). Taken together, β-TrCP1 appears to be a physiological substrate of SAG-UBCH10/UBE2S.

### SAG-βTrCP1 interaction has a complicated effect on βTrCP1 substrates

**Figure 5:**
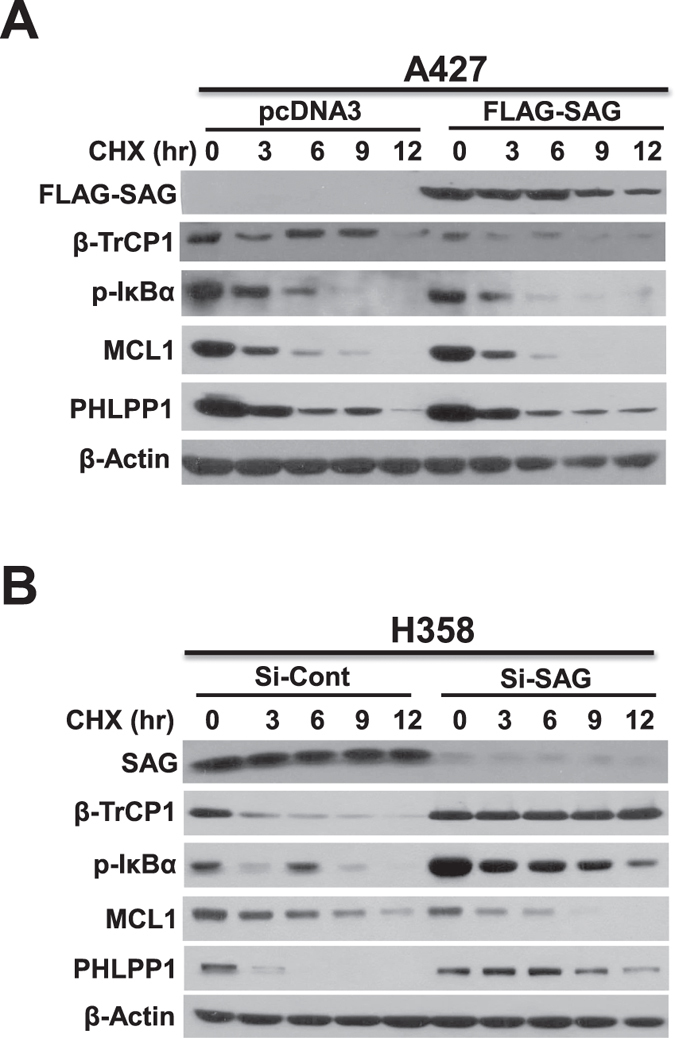
Effect of SAG manipulations on the levels of β-TrCP1 substrates. (**A**) A427 or **(B)** H358 cells were transfected with either plasmid expressing FLAG-SAG or siRNA targeting SAG, respectively, along with the vector or siCont oligonucleotide for 12 hrs. Cells were then cultured in fresh medium containing CHX and incubated for indicated time periods before being harvested for immunoblotting with indicated Abs.

Finally, we determined potential effect of SAG on the turnover of several known substrates of β-TrCP1. We focused on IκBα^34^, PHLPP1^35^, and MCL1^36^ after SAG manipulation for any changes in their protein half-lives. As shown in Fig. 5A and B, protein half-life of IκBα, like that of β-TrCP1, were shortened or extended upon SAG overexpression or silencing, respectively, suggesting that IκBα is likely a direct SAG substrate, as we previously shown^19^,^37^. In addition, although protein half-life of PHLPP1 had no change upon SAG overexpression, it is significantly extended upon SAG silencing, again suggesting that it is also likely a direct SAG substrate. In contrast, half-life of MCL1 had little change upon SAG overexpression, but was extended upon SAG silencing, suggesting that MCL1 is likely a direct substrate of β-TrCP1.

## Discussion

In this study, we made two novel findings: (1) there is a cross-talk between two CRLs, namely CRL5 and CRL1/SCF E3s. Specifically, CRL5 can inhibit SCF^βTrCP1^ E3 ligase activity by promoting β-TrCP1 ubiquitylation via an atypical K11 linkage for subsequent degradation, mediated by SAG. (2) SAG preferentially forms the complex with K11 E2s (UBCH10/UBE2C and UBE2S) to promote the formation of atypical polyubiquitylation chain via K11 linkage, whereas RBX1 exclusively forms the complex with K48 E2s (e.g. CDC34 and UBCH5C) to promote the formation of polyubiquitylation chain via typical K48 linkage. It is SAG or RBX1, via binding to different E2s, determines the linkage specificity in a manner independent of cullins.

Several E3 ubiquitin ligases have been previously shown to be involved in β-TrCP1 degradation, although detailed characterizations were lacking. Examples include SMURF2^38^ and SKP2^39^. Pursuit to our unexpected observations that the levels of SAG and β-TrCP1 were inversely correlated in mouse embryonic stem cells, in mouse pancreatic tissues and in multiple lung cancer lines, we reported here that β-TrCP1 is indeed a novel substrate of SAG-CUL5 or SAG-CUL1 E3s through UBCH10/UBE2S-mediated, K11-linked polyubiquitylation with the following lines of supporting evidence: (a) β-TrCP1 and SAG bind to each other under physiological conditions; (b) β-TrCP1 levels are reduced or increased upon SAG-CUL5 overexpression or SAG silencing, respectively; (c) β-TrCP1 protein half-life is shortened by SAG-CUL5 overexpression, but extended by SAG knockdown; (d) β-TrCP1 is subjected to polyubiquitylation by SAG-CUL5 or SAG-CUL1; (e) β-TrCP1 is accumulated upon expression of ubiquitin K11R mutant in U2-OS cells; and (f) β-TrCP1 is accumulated upon siRNA silencing of UBCH10 or UBE2S. Thus, β-TrCP1 joins a growing list of SAG substrates^16^. Given that β-TrCP1 is a substrate-recognizing subunit of SCF/CRL1 that regulates a variety of biological processes by promoting ubiquitylation and degradation of many key signaling molecules^2^,^22^, our study suggested that SAG-CRL5 can indirectly regulate these processes by counteracting the effect of SCF^β-TrCP1^. Our study, therefore, established a negative cross-talk network among CRLs. It is worth noting that SAG is not the substrate-recognizing subunit of CRL5 E3 ligase, direct binding of SAG-CUL5 complex with β-TrCP1 is most likely mediated by one of SOCS (Suppressor of cytokine signaling) box-containing proteins (for reviews, see Refs 40 and 41). Future study is directed to identify and characterize such a SOCS protein.

Although our mouse germline knockout studies revealed that *Rbx1* and *Sag* are not redundant and cannot compensate with each other during mouse embryonic development, as evidenced by embryonic lethality if either gene was disrupted^21^,^17^, biochemical difference between two members of RING family of CRL E3 ligase was previously unknown. In the present study, we provided convincing experimental data, using both *in vivo* and *in vitro* ubiquitylation assays and various ubiquitin mutants, clearly showed that SAG, by coupling with E2s UBCH10/UBE2C and UBE2S, promotes the formation of polyubiquitylation chain via K11 linkage, whereas RBX1, by coupling with E2 CDC34 or UBCH5C, catalyzes the formation of polyubiquitylation chain via K48 linkage. Moreover, we demonstrated that SAG-mediated polyubiquitylation of its substrates, such as β-TrCP1, p27 and Erbin via K11 linkage is E2-dependent and cullin-independent. We further demonstrated that β-TrCP1 is subjected to ubiquitylation and subsequent degradation by SAG-CUL1/5, but not by RBX1-CUL1, whereas p27 and Erbin can be ubiquitylated by either SAG-CUL1/5 or RBX-CUL1 via K11 or K48 linkage, respectively. This biochemical difference in selective E2 binding and substrate degradation can explain why *Sag* and *Rbx1* are not functionally redundant *in vivo*, particularly during mouse embryogenesis^15^,^17^.

A key feature of ubiquitin is its ability to form polymers, in which individual moieties are linked via one of seven Lys residues (Lys6, Lys11, Lys27, Lys29, Lys33, Lys48 or Lys63 linkages) or the N terminus (linear linkage) *in vivo* and *in vitro*^7^. The K48, K63, and K11 linkages have consistently been reported as the most abundant forms of polyUb in yeast and mammalian cells by mass spectrometry methods^42^,^43^. The cellular roles of Lys48- and Lys63-linked polyubiquitin have been extensively studied. While Lys48-linked polyubiquitin serves as a targeting signal for proteasomal degradation^44^, Lys63-linked ubiquitin chains are involved in cell signaling, initiate membrane trafficking events, and DNA damage response^45^. Although the cellular functions of Lys11-linked chains are less well understood, recent studies have, however, found the involvement of this linkage type in diverse cellular pathways, such as cell cycle^10^, endocytosis^46^, TNF signaling^47^, and WNT signaling^48^. Nevertheless, the best studied case for Lys11 linkage is catalyzed by APC/C (Anaphase-Promoting Complex/Cyclosome) E3 ligase complex, leading to rapid degradation of substrates during cell cycle progression, particularly at the mitosis^49^,^50^. The APC/C can utilize ‘priming’ E2 enzymes such as UBE2C/UBCH10 or UBE2D to decorate substrates with mono-ubiquitin and short ubiquitin chains^44^,^51^, and UBE2S then attaches ubiquitin to the already attached ubiquitin molecules, elongating polyubiquitin chains in a K11 linkage-specific manner^52^. In this study, we made a novel finding that SAG, on one hand, complexes with these two E2s, and on the other hand, binds to CUL5 or CUL1 to promote polyubiquitylation of β-TrCP1 via K11 for targeted degradation. Thus, it is likely that SAG-CRLs and APC/C would compete with each other for selective binding of K11 E2s in cell/tissue context- and stress-dependent manners to precisely regulate complicated biological processes. Our study, therefore, established yet another cross-talk between CRLs and APC/C, two largest E3 ubiquitin ligases via competing for K11 E2s.

Finally, we examined potential effect of SAG manipulation on the turnovers of several known substrates of β-TrCP1. This appears to be a complicated analysis, given that fact that both SAG and β-TrCP1 are E3 ligases that could target the same substrates^16^,^53^, while β-TrCP1 itself is yet a direct substrate of SAG. It is anticipated that the turnover of a given substrate should be accelerated or reduced upon SAG overexpression or depletion, respectively, if it is a direct SAG substrate, whereas the opposite would be true, if it is a direct substrate of β-TrCP1. In our experimental setting, we found IκBα and PHLPP1 are likely direct substrates of SAG, whereas MCL1 is more subjected to targeted degradation by β-TrCP1. Thus, the net biological outcome will be determined by whether the substrates are targeted by SAG directly or indirectly via β-TrCP1, as well as whether β-TrCP1 is directly targeted by SAG in a given cell line. The biological effect is, therefore, truly cell context dependent. Our previous studies as well as the studies from other groups have clearly demonstrated that SAG is a *bona fide* anti-apoptotic protein (for review, see ref. 16) through directly targeting its substrates, likely including β-TrCP1.

## Materials and Methods

### Cell culture

Human embryonic kidney HEK293T and various human lung cancer cells were purchased from American Type Culture Collection. Establishment of U2OS cells expressing shUb-Ub (WT), -Ub (K11R), -Ub (K48R), and -Ub (K63R) were described previously^33^. Mouse embryonic stem cells (AB2, wild type and AB1, Sag-null) were cultured as described^19^. HEK293, U2OS-shUb-Ub (WT), -Ub (K11R), -Ub (K48R), and -Ub (K63R) were grown in DMEM with 10% FBS; A427 and H358 were grown in RPMI-1640 with 10% FBS. All cell lines were tested and free of mycoplasma contamination.

### Transfection

Transfections of plasmids or siRNAs (synthesized by Dharmacom, Lafayette, CO) were performed using the lipofectamine 2000 (Invitrogen), following the manufacturer’s instruction. The sequences of siRNAs targeting SAG, RBX1, CUL-1, CUL-5, UBCH10, UBE2S, UBCH5C and scrambled siCont are as follows: siSAG: 5′-CAGAAGGCTCATGGTAAAACATC-3′; siRBX1: 5′-GACTTTCCCTGCTGTTA CCTAA-3′; siCUL1: 5′-GGTCGCTTCATAAACAACA-3′; siCUL5: 5′-GTCTCA CTTCCTACTGAACTG-3′; Si-UBCH10: 5′-CCUGCAAGAAACCUACUCAUU-3′^54^, Si-UBCH5C: 5′-GAUGAUGUAAAGUUCGAAAGAUU-3′^55^; si-Cont: 5′-AUUG UAUGCGAUCGCAGAC-3′. The Si-UBE2S (siRNA pools) was purchased from Santa Cruz Biotechnology (Santa Cruz, CA).

### Co-immunoprecipitation and immunoblotting

To immunoprecipitate endogenous proteins, whole cell extracts were pre-cleared with normal IgG-AC (Santa Cruz) followed by overnight incubation at 4 °C with indicated antibodies. To immunoprecipitate exogenously expressed FLAG-tagged or HA-tagged proteins, the pre-cleared cell lysates were incubated with FLAG (Sigma) or HA antibody (Roche) for 3 hrs followed by incubation with protein A&G beads (Santa Cruz) at 4 °C overnight with rotation. The beads were washed three times with lysis buffer, and the immunoprecipitation complexes were subjected to SDS-PAGE^56^.

Whole-cell lysates were prepared and subjected to immunoblotting analysis using antibodies against SAG [11], ROC1 [42], HA (Roche), FLAG and actin (Sigma), CUL-5, CUL-1, UBE2C, CDC34 (Santa Cruz), UBE2S (Abcam), UBCH5C and β-TrCP (Cell Signal), p27 (BD phamingen), and FBXL3 and FBXL11 (Abcam).

### RT-PCR

Cells were transfected with plasmid DNA or siRNA oligoes for 48 hrs, followed by total RNA isolation, using RNAzol, and RT-PCR analysis. The primer sequences for βTrCP1 and GAPDH are as follows: β-TrCP1-F: 5′-TGTGGCC AAAACAAAACTTGCC-3′ and β-TrCP1-R: 5′-ATCTGACTCTGACCACTGCTC-3′; and GAPDH-F: 5′-GCCAAAAGGGTCATCATCTC-3′ and GAPDH-R: 5′-GTAGAG GCAGGGATGATGTTC-3′.

### The *in vivo* ubiquitylation

To determine ubiquitylation of β-TrCP1, β-TrCP1ΔF, p27 or Erbin, few indicated cell lines were co-transfected with plasmids encoding each protein, along with various ubiquitin constructs. Cells were lysed in 6 M guanidinium denaturing solution, as described previously^26^. Polyubiquitylated substrates were purified by Ni-bead pull-down and detected by IB using respective antibbodies.

### The *in vitro* ubiquitylation

HA-β-TrCP1 or HA-β-TrCP1ΔF was precipitated from 293 cells transfected with either encoding plasmid, and eluted with 3xHA peptide (Sigma), serving as the substrates; FLAG-tagged SAG-CUL5, RBX1-CUL1 was pulled down by FLAG bead (Sigma) after transfection into 293 cells, serving as the source of E3. The reaction was carried out at 30 °C for 1 hr by constant mixing in 30 μL reaction buffer (40 mM Tris-HCl, pH 7.5, 2 mM DTT, 5 mM MgCl_2_) in the presence of commercially purchased E1, E2s (UBCH10, UBE2S or CDC34, UBCH5C), and ubiquitin (all from Boston Biochem), and above purified substrates and E3s. Poly-ubiquitinated substrates was resolved by SDS-PAGE and detected by IB with anti-HA Ab.

## Additional Information

**How to cite this article**: Kuang, P. *et al*. SAG/RBX2 E3 ligase complexes with UBCH10 and UBE2S E2s to ubiquitylate β-TrCP1 via K11-linkage for degradation. *Sci. Rep.* **6**, 37441; doi: 10.1038/srep37441 (2016).

**Publisher's note:** Springer Nature remains neutral with regard to jurisdictional claims in published maps and institutional affiliations.

## Supplementary Material

Supplementary Materials
