# Supplementary material for: SAG/RBX2 E3 ligase complexes with UBCH10 and UBE2S E2s to ubiquitylate β-TrCP1 via K11-linkage for degradation
Source: Sci Rep. 2016 Dec 2;6:37441. doi: 10.1038/srep37441 (PMC5133542; doi:10.1038/srep37441)

SAG/RBX2 E3 ligase complexes with UBCH10 and UBE2S E2s to ubiquitylate β-TrCP1 via K11-linkage for degradation

Peng Kuang[1,2], Mingjia Tan[2], Weihua Zhou[2], Qiang Zhang[2], and Yi Sun[2-4]

[1]Department of Internal Medicine, Beijing University School of Medicine, 38 Xueyuan Road, Beijing, 100191, China

[2]Division of Radiation and Cancer Biology, Department of Radiation Oncology, University of Michigan, 4424B MS-1, 1301 Catherine Street, Ann Arbor, MI 48109, USA.

[3]Institute of Translational Medicine, Zhejiang University School of Medicine, Hangzhou, Zhejiang, People's Republic of China.

[4]Collaborative Innovation Center for Diagnosis and Treatment of Infectious Diseases, Zhejiang University, Hangzhou, P.R. China;

[#]Corresponding author: email: sunyi@umich.edu or yisun@zju.edu.cn

Running title: SAG ubiquitylates βTrCP for degradation

Key words: β-TrCP, CRL5, RBX1, SAG, UBE2S, and UBCH10

Supplemental Figure legends:

Figure S1. Inverse relationship between SAG and βTrCP1, and their binding to cullins.

(A) Inverse correlation between SAG and βTrCP: Cell lysates were prepared from various lung cancer cell lines, followed by IB using indicated Abs.

(B) SAG-βTrCP1-cullins form the complex: The 293T cells were transfected with FLAG-tagged CUL1 or CUL5, followed by IP with anti-FLAG Ab or IgG control, and IB with indicated Abs.

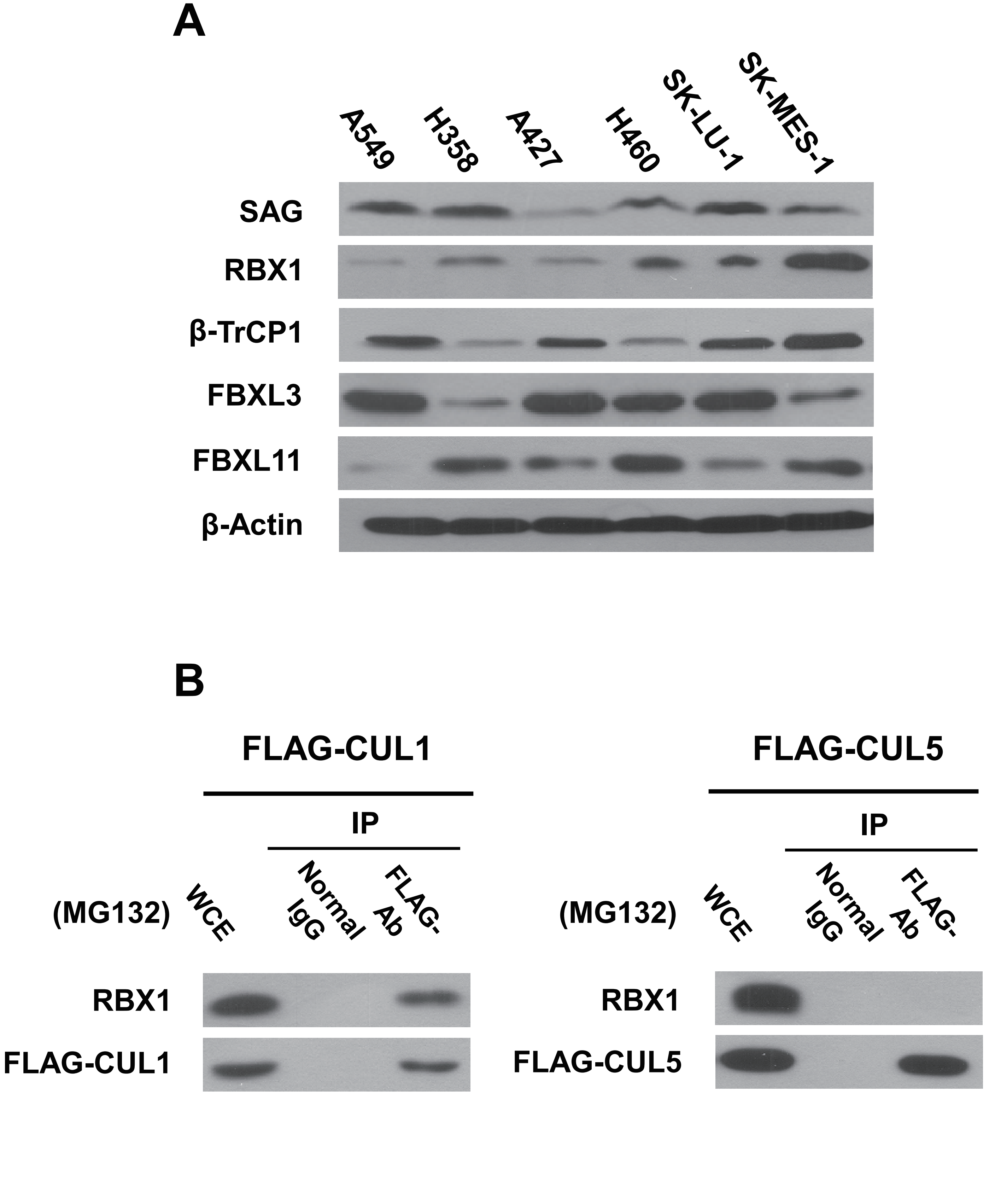
A
A549
H358
A427
H460
SK-LU-1
SK-MES-1
SAG
RBX1
β-TrCP1
FBXL3
FBXL11
β-Actin
B
FLAG-CUL1
IP
(MG132)
WCE
Normal IgG
FLAG-Ab
RBX1
FLAG-CUL1
FLAG-CUL5
IP
(MG132)
WCE
Normal IgG
FLAG-Ab
RBX1
FLAG-CUL5

Figure S2. SAG negatively regulates β-TrCP1 protein level.

(A) SAG-CUL5 reduces βTrCP1 level: The 293T cells were transfected with indicated plasmids. Whole cell extracts were analyzed by IB with indicated antibodies. FLAG-1: CUL1 or CUL5; FLAG-2, SAG or RBX1.

(**B&C**) Accumulation of βTrCP1 and p27: Two indicated lung cancer lines were treated with various concentrations of MLN4924 for 24 hrs, followed by IB, using indicated Abs.

(**D&E**) SAG-CUL5 shortens the half-life of βTrCP1 and βTrCP1ΔF: After transfection with indicated plasmids for 12 hrs, cells were switched to fresh medium (10% FBS) containing cycloheximide (CHX) and incubated for indicated time periods before being harvested for IB using indicated Abs.

(**F**) Manipulation of SAG-CUL5 has no effect on β-TrCP mRNA level. A427 cells were transfected with indicated plasmid or siRNA oligoes. Forty-eight hrs post-transfection, total RNA was isolated and subjected to RT-PCR analysis for β-TrCP mRNA levels.

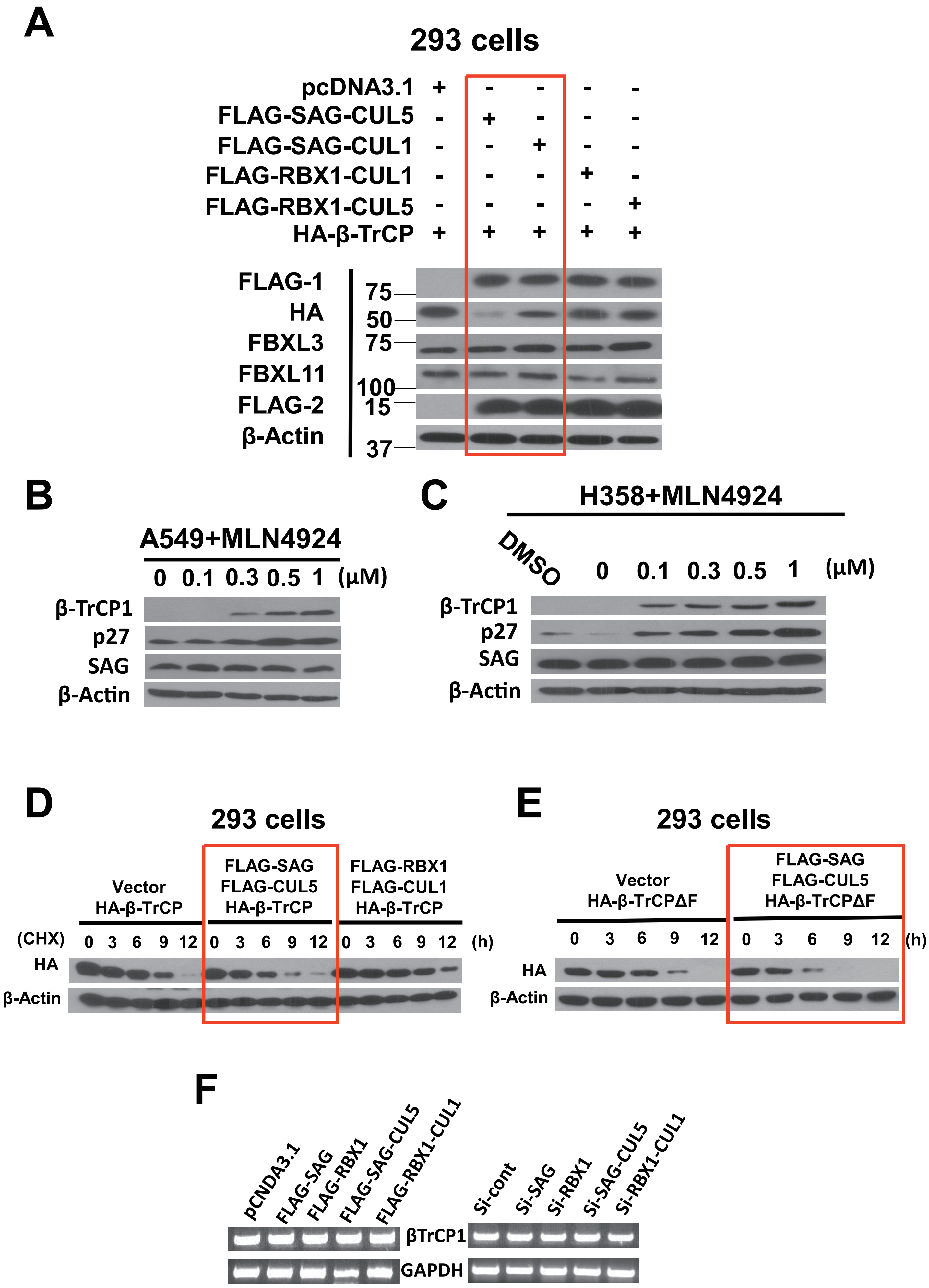
A
293 cells
pcDNA3.1
FLAG-SAG-CUL5
FLAG-SAG-CUL1
FLAG-RBX1-CUL1
FLAG-RBX1-CUL5
HA-β-TrCP
FLAG-1
HA
FBXL3
FBXL11
FLAG-2
β-Actin
75
50
75
100
15
37
B
A549+MLN4924
0 0.1 0.3 0.5 1 (μM)
β-TrCP1
p27
SAG
β-Actin
C
H358+MLN4924
DMSO
0 0.1 0.3 0.5 1 (μM)
β-TrCP1
p27
SAG
β-Actin
D
293 cells
Vector
HA-β-TrCP
FLAG-SAG
FLAG-CUL5
HA-β-TrCP
FLAG-RBX1
FLAG-CUL1
HA-β-TrCP
(CHX) 0 3 6 9 12 0 3 6 9 12 0 3 6 9 12 (h)
HA
β-Actin
E
293 cells
Vector
HA-β-TrCPΔF
FLAG-SAG
FLAG-CUL5
HA-β-TrCPΔF
0 3 6 9 12 0 3 6 9 12 (h)
HA
β-Actin
F
pCNDA3.1
FLAG-SAG
FLAG-RBX1
FLAG-SAG-CUL5
FLAG-RBX1-CUL1
Si-cont
Si-SAG
Si-RBX1
Si-SAG-CUL5
Si-RBX1-CUL1
βTrCP1
GAPDH

Figure S3. SAG-CUL5, but not RBX1-CUL1, promotes the ubiquitylation of βTrCP1 via K11 linkage.

(A) SAG-CUL5 promotes polyubiquitylation of β-TrCP1 and β-TrCP1ΔF *in vivo*: H1299 cells were transfected with indicated plasmids, lysed under denatured condition by 6M guanidinium solution, followed by Ni-bead pull-down. Washed beads were boiled and subjected to IB, along with whole cell lysates, using indicated Abs.

(B-E) SAG/CUL5 promotes poly-ubiquitylation of β-TrCP1 via K11 linkage: H1299 (B&D) or 293T (C&E) cells were cotransfected with indicated plasmids alone or in combination, along with ubiquitin and various ubiquitin mutants. Whole cell extracts and Ni-NTA affinity purified fractions were analyzed by IB using indicated Abs.

Kuang et al., Figure S3

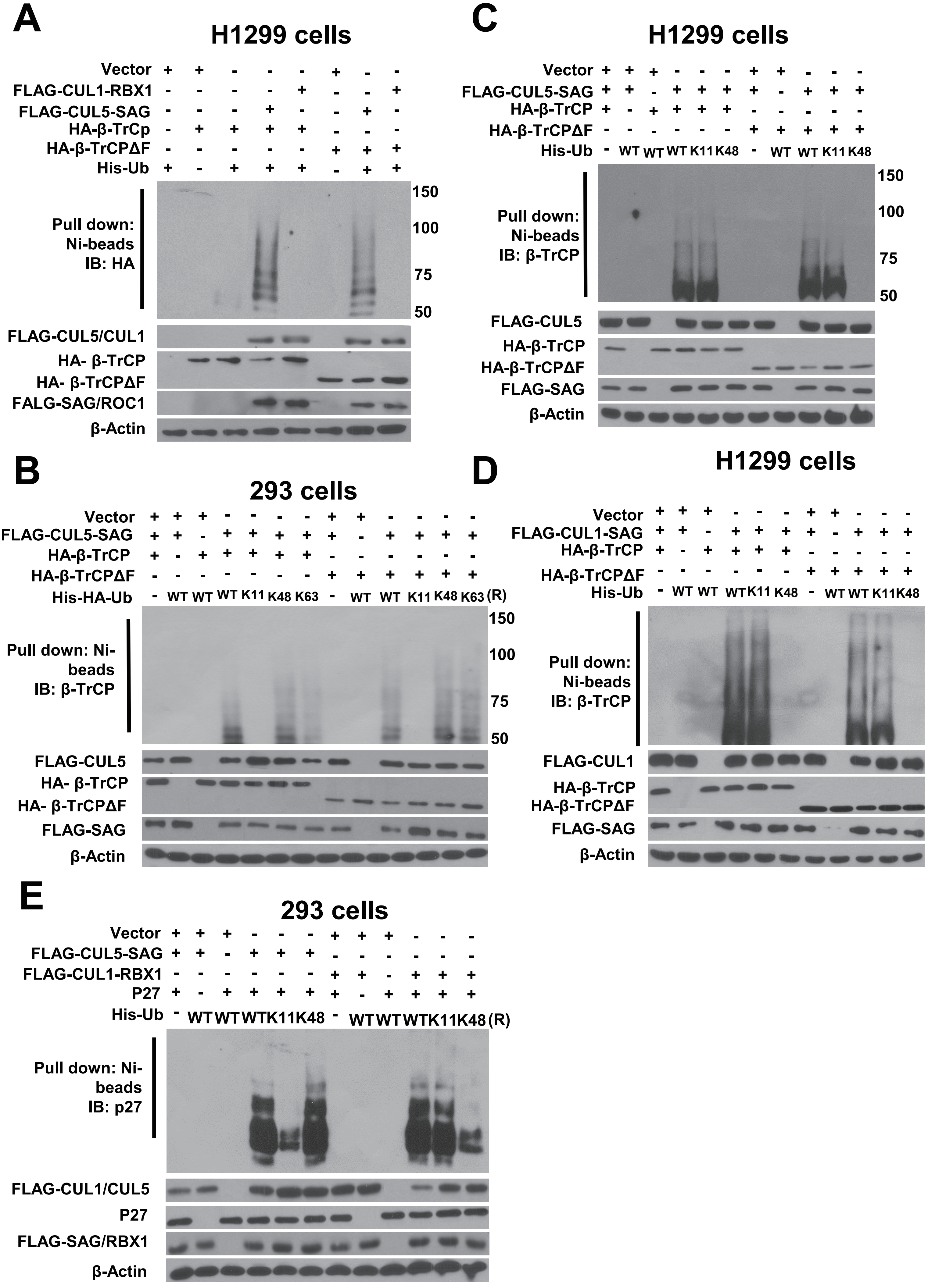

Figure S4. SAG and RBX1 form the complex with different E2s.

(A) H1299 cells were transfected with FLAG-SAG or FLAG-RBX1, followed by IP using FLAG Ab or normal IgG control, and IB with indicated Abs.

(B&C) Whole cell extracts (WCE) from A427 cells were subjected to IP and IB, or directly subjected to IB with indicated Abs.

(**D**) The 293T cells were transfected with indicated plasmids alone or in combination, and subjected to in vitro ubiquitylation as described in M&M. The reaction mixture was then loaded onto PAGE gel for IB using anti-Erbin Ab.

Kuang et al., Figure S4

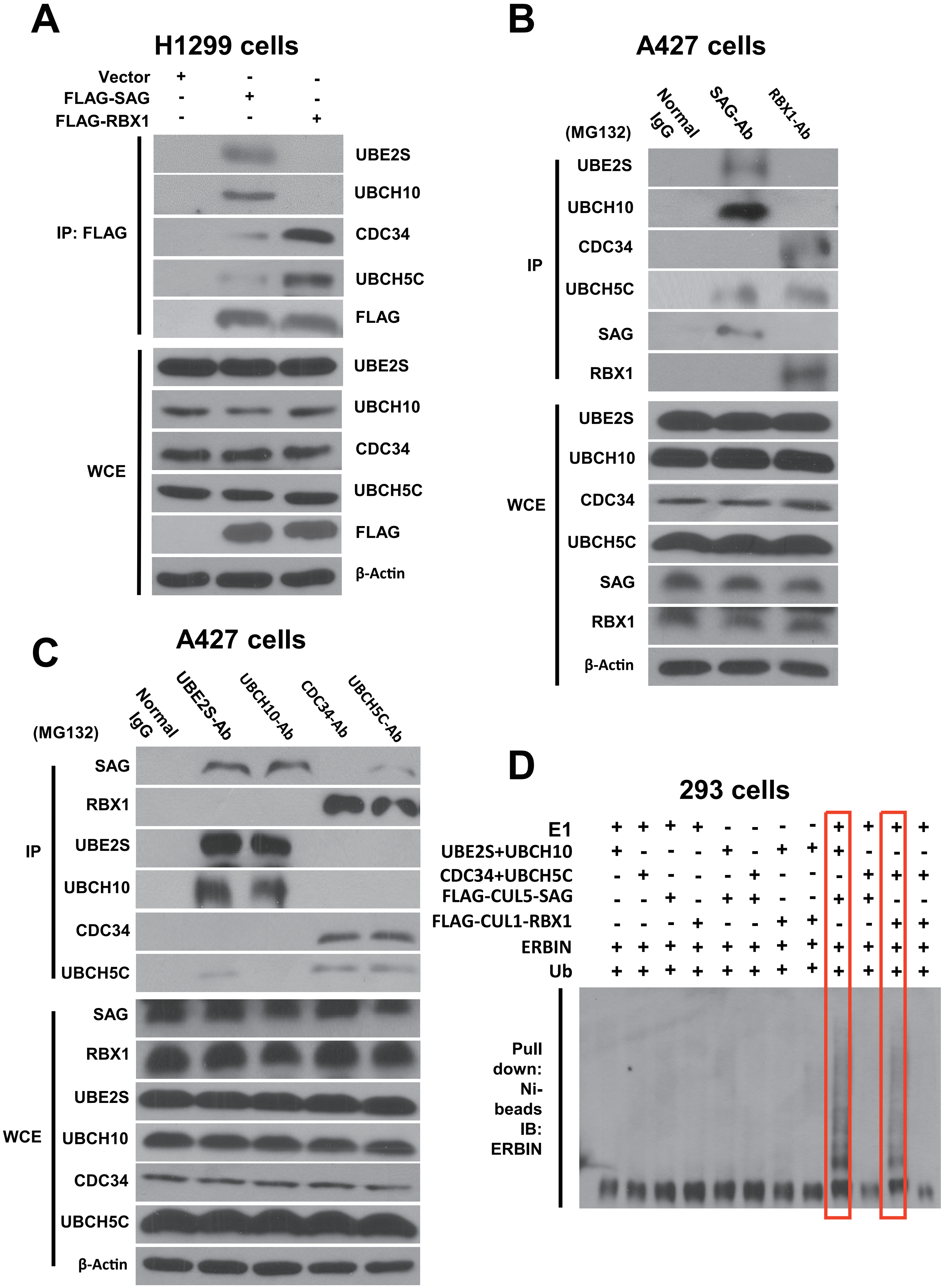

Supplement: Supplementary Materials [file srep37441-s1.pdf]
